# When less conditioning provides better estimates: overcontrol and endogenous selection biases in research on intergenerational mobility

**DOI:** 10.1007/s11135-021-01310-8

**Published:** 2022-01-04

**Authors:** Michael Grätz

**Affiliations:** 1 Swedish Institute for Social Research (SOFI), Stockholm University, Stockholm, Sweden; 2 Institut des sciences sociales, University of Lausanne, Quartier UNIL-Mouline, Bâtiment Géopolis, CH- 1015 Lausanne, Switzerland

**Keywords:** Causality, Directed acyclic graphs, Endogenous selection bias, Intergenerational mobility, Overcontrol bias

## Abstract

The counterfactual approach to causality has become the dominant approach to understand causality in contemporary social science research. Whilst most sociologists are aware that unobserved, confounding variables may bias the estimates of causal effects (omitted variable bias), the threats of overcontrol and endogenous selection biases are less well known. In particular, widely used practices in research on intergenerational mobility are affected by these biases. I review four of these practices from the viewpoint of the counterfactual approach to causality and show why overcontrol and endogenous selection biases arise when these practices are implemented. I use data from the German Socio-Economic Panel Study (SOEP) to demonstrate the practical consequences of these biases for conclusions about intergenerational mobility. I conclude that future research on intergenerational mobility should reflect more upon the possibilities of bias introduced by conditioning on variables.

## Introduction

In the last decades, research practices in the social sciences have seen major changes due to the emergence of the counterfactual approach to causality as the primary way to understand causality in social science research (Angrist and Pischke 2009; Morgan and Winship 2015; Pearl 2009; Rubin 2005; VanderWeele 2015). An important aspect of the counterfactual approach to causality is the increased importance for researchers to spell out and to justify the assumptions needed to interpret statistical estimates as causal effects because of the possibility of omitted variable bias.

Whilst most sociologists are aware of the threat to the identification of causal effects caused by omitted variable bias due to unobserved, confounding variables, the dangers of overcontrol and endogenous selection biases have received less attention. With a focus on endogenous selection bias, Elwert and Winship (2014) discussed the issues arising in this context but these insights have not yet fully translated into research practices in all research fields within the social sciences. The aim of this article is to apply the insights arising from the counterfactual approach to causality to the study of the intergenerational transmission of advantage.

Researchers studying the intergenerational transmission of advantage are not always explicit about whether they are interested in estimating causal effects or in providing descriptive estimates. Torche (2015) argued that the main aim of research on intergenerational mobility should be to provide descriptive estimates of the similarity in resources between parents and their offspring. These descriptive estimates provide important yardsticks about the degree of equality of opportunity in societies. There are, however, several widely used practices in research on intergenerational mobility that go beyond providing descriptive estimates, i.e. these approaches require a causal interpretation of estimates.^1^ There are important insights that can be gained from applying the counterfactual approach to causality to understand the consequences of employing these research practices. These insights have not yet been fully acknowledged by many researchers studying intergenerational mobility. Torche (2015) touched on some of the issues that arise if we apply the counterfactual framework of causality to the study of intergenerational mobility, for instance the interpretation of underlying mechanisms based on path-analytical models. However, there are other widely used practices in research on intergenerational mobility that are problematic from the perspective of the counterfactual approach to causality.

I discuss four widely used practices in research on intergenerational mobility that lead to overcontrol and endogenous selection biases. I illustrate the practical consequences of these biases, to the degree that this is possible, using data from the German Socio-Economic Panel Study (SOEP), a data set that is widely used to study intergenerational mobility in Germany (e.g., Grätz and Pollak 2016; Hertel 2017; Müller and Pollak 2004). The aim of my article is to bring the issues of overcontrol and endogenous selection biases to the attention of researchers who work on intergenerational mobility.

## Background and theoretical considerations

### Omitted, overcontrol, and endogenous selection biases in research on intergenerational mobility

Under which conditions can (conditional or unconditional) associations between parental and child characteristics be interpreted as causal effects? There are (at least) three sources of bias making such an interpretation challenging. Bias is the failure to correctly estimate the causal effect of interest. The first and most widely known type of bias is omitted variable bias. Omitted variable bias occurs if unobserved variables affect both parental and child characteristics. Researchers studying the intergenerational transmission of advantage are nowadays well aware of the threat of omitted variable bias to the identification of causal effects.

The present article discusses two other, less widely known types of bias: overcontrol bias and endogenous selection bias. Omitted variable bias arises if we do not condition on variables that we should condition upon. Contrary to that, overcontrol and endogenous selection biases arise if we condition on variables that we should not condition upon.^2^ These two biases are well known and discussed in the causality literature (Elwert and Winship 2014). However, they still arise in many (recent) empirical analyses in research on intergenerational mobility.

These two biases can be defined in the following ways:

*Overcontrol Bias.* Conditioning on a variable that lies on the causal path between the two variables of interest, i.e. the treatment and the outcome, leads to overcontrol bias in the estimation of the total causal effect (Elwert and Winship 2014).^3^

*Endogenous Selection Bias.* Conditioning on a collider (or a descendant of a collider) leads to endogenous selection bias in the estimation of the effect of a treatment on an outcome. A collider is a variable that is affected by two variables: (1) the treatment or a variable associated with the treatment and (2) the outcome or a variable associated with the outcome (Elwert and Winship 2014:31).^4^ Collider variables are path specific, i.e. a variable may be a collider on one path and a non-collider on another path. Crucially, if a variable is a collider on the path linking the treatment to the outcome, conditioning on this variable leads to endogenous selection bias. Conditioning on a collider leads to endogenous selection bias because it opens up a non-causal path between the treatment and the outcome and therefore induces a spurious association between the treatment and the outcome.

**Fig. 1:**
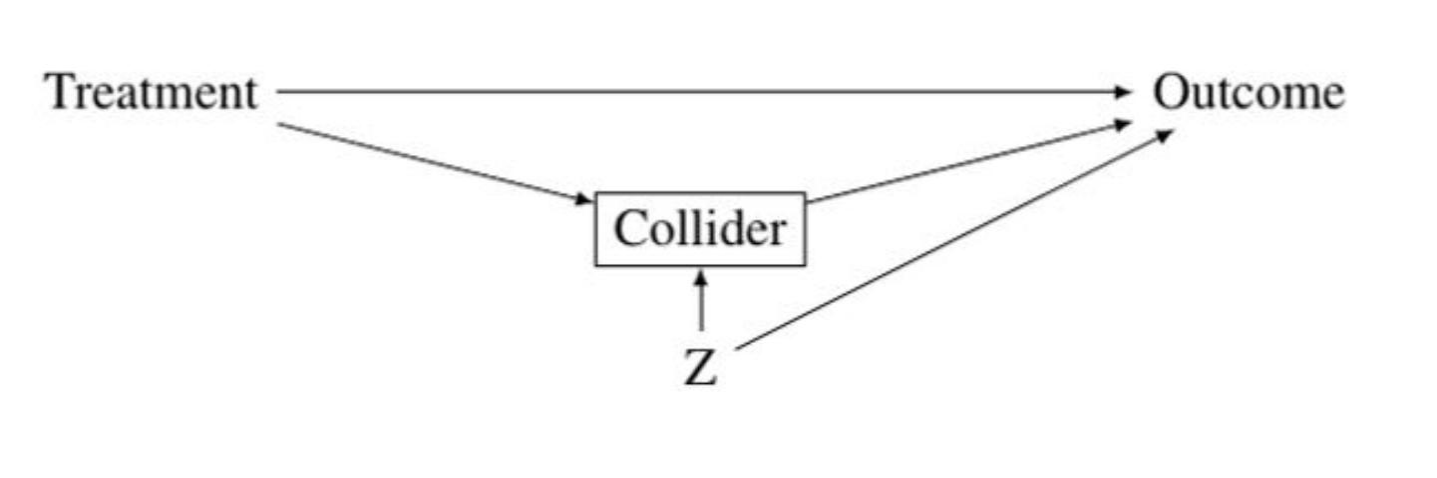
Illustration of endogenous selection bias in a mediation analysis

In the four research practices discussed in this article, the collider variable takes the form of a mediator. That is to say the analysis aims to decompose the total causal effect into an indirect effect mediated by the mediator (the collider) and a direct effect that is not mediated by the mediator.^5^ Figure 1 illustrates this set up. The variables included in the vector Z affect both the collider and the outcome and lead therefore to endogenous selection bias in estimating the direct effect of the treatment on the outcome (Breen 2018). The only way to correctly estimate the direct effect of the treatment on the outcome when conditioning on a collider is by also conditioning on all variables that relate the collider to the outcome—an enterprise that is impossible if some of the variables included in Z are unobserved.

A particular problematic version of endogenous selection bias arises when Z is itself affected by the treatment. Acharya, Blackwell, and Sen (2016) called this special type of endogenous selection bias intermediate variable bias. In this situation, even controlling for the confounding variables included in Z introduces a bias, as the direct effect of the treatment on the outcome will then be estimated with overcontrol bias.

In the following, I discuss four widely used practices in research on the intergenerational transmission of advantage that have in common that they lead to overcontrol and endogenous selection biases. I discuss these widely used research practices, the overcontrol and endogenous selection biases arising through them, and the implications of these biases for our conclusions about intergenerational mobility. The focus of my discussion is on the causal models researchers need to entertain in order to justify the application of these practices. My impression is that many researchers are not aware of the assumptions underlying these research practices and of the overcontrol and endogenous selection biases that arise if they are employed.

Throughout the following discussion, I use Directed Acyclic Graphs (DAGs) to motivate the issues at stake. A DAG is a graphical way to display causal relationships. A researcher draws a DAG based on her/ his believes about the causal relationships among variables. Therefore, constructing a DAG is a theoretical, and not an empirical, task. DAGs are a convenient and easy way to visualize theoretical assumptions and are an important toolbox of counterfactual thinking (Elwert 2013; Elwert and Winship 2014; Pearl 2009). DAGs make it possible to discuss the assumptions underlying empirical analyses in an intuitive way. For instance, Breen (2018) used DAGs to discuss endogenous selection bias (which he referred to as “collider bias”) arising in studies analysing intergenerational mobility across three generations.

DAGs visualize hypothesized causal effects through arrows. An arrow pointing from one variable to another represents a causal effect. A → B indicates that A causally affects B.^6^ I follow the convention in the literature to indicate variables that are conditioned on by a rectangular box (Elwert 2013). In addition, I do indicate unobserved variables in the models by the term “Unobserved”. Even though they are only indicated through one node, there usually are several possible unobserved variables in each graph so it is best to think about every node with the term “Unobserved” as indicating a vector of unobserved variables that can confound the relationships of interest. The influence of unobserved variables (omitted variable bias) is a general problem, as there is always the danger that unobserved variables confound the estimates of intergenerational mobility.

The DAGs I use are certainly oversimplifications of reality. I use them to illustrate the problems of overcontrol and endogenous selection biases. More complicated DAGs, describing more complex data-generating processes, could be drawn. However, the DAGs I use are enough to illustrate my points. One aim of my article is to motivate researchers to draw DAGs of the processes they are interested in before conducting empirical analyses.

**Fig. 2:**
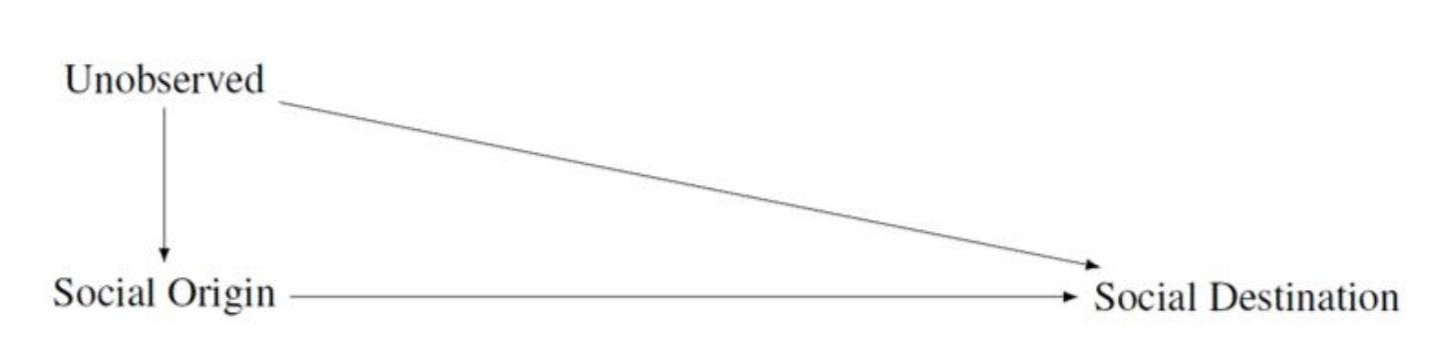
The basic model underlying the analysis of intergenerational mobility. Social origin: measured, for instance, via parental education, income, or occupation. Social destination: measured, for instance, via child education, income, or occupation. Unobserved is a vector of unobserved variables

 Figure 2 provides a DAG of the effect of parental resources (social origin) on child outcomes (social destination). This bivariate relationship is the main focus of research on intergenerational mobility. The figure also indicates that there may be unobserved variables confounding the relationship between social origin and social destination. This is why estimates of intergenerational mobility have to be interpreted associationally and cannot be interpreted causally (Björklund and Jäntti 2020; Torche 2015). Examples of such unobserved variables can be, among many others, innate parental abilities and personality traits.

 The research practices I discuss below go beyond describing the bivariate relationship portrayed in Fig. 2. They require researchers to have in mind specific causal models of the relationships they are interested in studying. These models are also called data-generating processes (Elwert and Winship 2014) and are essentially the theoretical assumptions underlying the empirical analyses. These models, which I discuss in the following, can lead to overcontrol and endogenous selection biases.

### Research practice 1: conditioning on multiple dimensions of social origin

The first research practice that introduces overcontrol and endogenous selection biases is the practice to include several measures of social origin, for instance parental education, occupation, status, income, social capital, cultural capital, and wealth, in the same model as independent variables. One of the founders of empirical sociology Paul F. Lazarsfeld (1939) argued that different measures of social origin could be used interchangeably. In the following decades the status attainment approach became the dominant approach to the study of the intergenerational transmission of advantage. The status attainment approach was based on path-analytical models in which the different indicators of family background were thought to be exogenously determined and disentangled (Blau and Duncan 1967; Featherman and Hauser 1978; Sewell et al. 1969; Sewell and Hauser 1975).

 The status attainment tradition influences contemporary practices in research on intergenerational mobility. Some contemporary researchers argue that different dimensions of family background have different effects on respondent’s educational and occupational attainment (Bukodi and Goldthorpe 2013; Erola et al. 2016; Hällsten and Pfeffer 2017; Hällsten and Thaning 2018; Jæger 2007; Jæger and Holm 2007; Mood 2017; Pfeffer 2018; Wong 1998). These claims are made using models in which different indicators of social origin are entered in the same model and in which the results show that these different indicators have statistically significant and substantively meaningful associations with the outcome (some measure of children’s social destination) after conditioning on the other indicators of social origin.

**Fig. 3:**
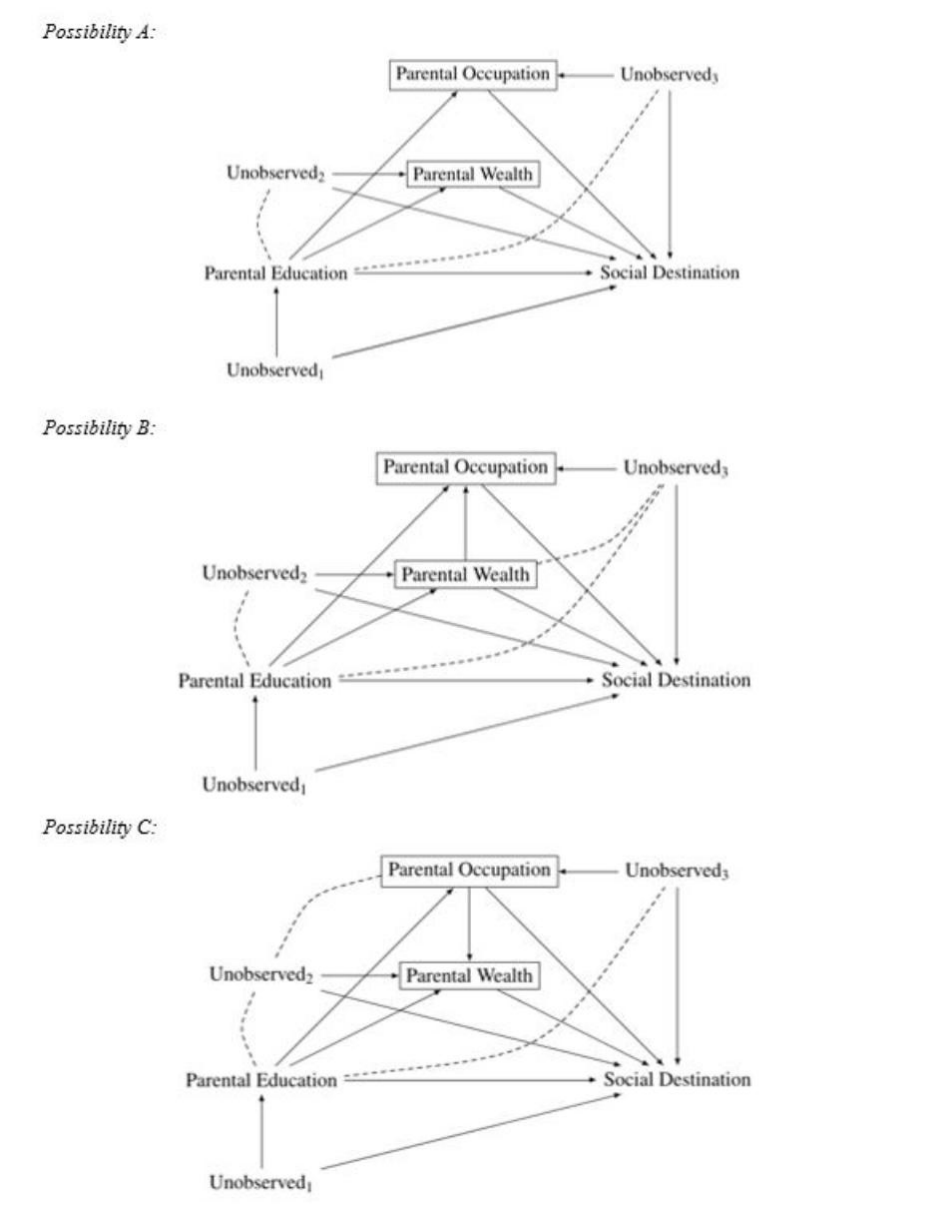
Conditioning on multiple dimensions of social origin. Social destination: measured, for instance, via child education, income, or occupation. Unobserved_1_, Unobserved_2_, and Unobserved_3_ are vectors of unobserved variables

 In Fig. 3, I illustrate the causal model, i.e. the data-generating process, that researchers need to entertain if they include measures of parental education, occupation, and wealth into the same model. The same logic applies if more indicators of social origin (parental income, cultural capital, social capital, and others) are added to the model but the three variables I choose are enough to illustrate my point.

 A first insight from the DAG in Fig. 3 is that the estimate of the total causal effect of parental education on social destination is biased if the same model controls for parental occupation and parental wealth. Most researchers would argue that parental occupation is a variable lying on the causal path between parental education and social destination and should therefore not be controlled for when researchers are interested in estimating the total causal effect of parental education on children’s outcomes (social destination). Parental wealth can also be a variable lying on the causal path between social origin and social destination, although if the parents inherited large parts of their wealth from their own parents (the grandparents from the perspective of the children) before completing their education, parental wealth could also precede parental education. Estimating a model that controls for parental education and parental occupation and comparing these coefficients is misleading, as the total effect of parental occupation is compared to the direct effect of parental education conditional on parental occupation. It would, however, be necessary to compare the total effect of parental education to the total effect of parental occupation to decide which indicator of social origin has a stronger effect on social destination.

 Whilst probably most researchers agree that parental education precedes parental occupation (and, to a large part, parental wealth), the situation is less clear with respect to other measures of social origin. When entering parental occupation and parental wealth in the same model researchers have to assume that they are not affecting each other (Possibility A in Fig. 3). But parental wealth and parental occupation could directly affect each other (Possibility B and Possibility C in Fig. 3). What is more, it is difficult to make an argument that parental wealth precedes or proceeds parental occupation.

 On the one hand, parental occupation can precede parental wealth, as certain occupations will have higher salaries and allow men and women therefore to accumulate more wealth. On the other hand, men and women can have (inherited) wealth before deciding which occupation to take. A large amount of inherited wealth may allow men and women to choose occupations with lower financial returns and a lower socioeconomic status. For that reason, parental wealth can also precede parental occupation.^7^

 Because the relationship between parental wealth and parental occupation is theoretically ambiguous, it is best not to control for the other variable in the same model and to compare the estimates obtained using separate models. Alternatively, researchers should model the process by which parental wealth and parental occupation influence each other to be able to identify the causal effects of parental wealth and parental occupation on social destination. Or they should decompose parental wealth into different components, one inherited wealth, which may come causally prior to parental occupation, and one acquired wealth, which may come later.^8^ In any case, careful thinking about the causal order of different indicators of social origin is required and we should not compare the total effect of one indicator to the direct effect of another indicator.

 The arguments above apply if we are interested in comparing the effects of different measures of social origin. Researchers could also be interested in estimating the joint effect of the different indicators of social origin and then they can be entered in the same model. However, still, the effect strengths of the different indicators cannot be compared to each other. Nevertheless this is what often happens in research on intergenerational mobility. For instance, van de Werfhorst (2018, 2019) estimated the effects of reforms in tracking on the association between two indicators of social origin (parental occupation and parental education) and children’s academic performance. These studies conditioned on both indicators of social origin in the same model. The findings of no change in the association between parental and child education, which are different from the findings of a change in the association between parental occupation and child education, suffer from overcontrol bias.

 To sum up the discussion of this research practice, I argue that including several indicators of social origin in the same model as independent variables does lead to overcontrol bias in estimating the total effects of these indicators on social destination. This viewpoint is clearly at odds with current practices in stratification research. Mood (2017:282) justified current practices by claiming that “from the child’s viewpoint, parental education, occupation, and income are contemporaneous”. There are certainly reasons for taking such a view but it cannot be maintained if we are interested in knowing what the total causal effect of increasing parental education on children’s outcomes is. However, this is the policy-relevant question, as it is difficult to imagine a policy that raises parental education but that does neither affect parental occupation nor parental income (or wealth). Controlling for occupation and income (or wealth) at the parental level when estimating the total effect of parental education on social destination introduces overcontrol bias precisely because a high level of education allows parents to have a high level of income (wealth) and a high level of occupation, which then may be beneficial for their offspring.

 The issue of overcontrol bias is, however, not the only bias that can arise in Fig. 3. Conditioning on several indicators of social origin can also lead to endogenous selection bias. This is the case because parental occupation (or wealth) is a collider variable on the path from parental education to social destination. Conditioning on parental occupation (or wealth) therefore introduces a spurious association between parental education and any unobserved variables that affect both parental occupation (or wealth) and social destination. This spurious association is indicated by a dashed line between Unobserved_2_ (and Unobserved_3_) and parental education in Fig. 3. There are plenty of plausible candidates for such variables, including parental interest to foster their children’s development of cognitive and noncognitive skills, their education, and their labor market careers. Depending on which of the three possibilities indicated in Fig. 3 applies, there will also be endogenous selection bias when estimating the effect of parental occupation (or wealth) on social destination conditional on parental wealth (or occupation).^9^

### Research practice 2: conditioning on father’s and mother’s characteristics

 The second research practice that I analyse, including separate characteristics of mothers and fathers in the same model, is directly related to the first one. This practice has received a lot of attention in research on intergenerational mobility, as it is theoretically appealing. Researchers have argued that not including maternal along paternal characteristics into the same models of intergenerational mobility provides a misrepresentation of intergenerational mobility (Beller 2009; Bloome and Western 2011; Buis 2013; Jæger 2007; Kalmijn 1994; Korupp et al. 2002; Marks 2008). Models that include both maternal and paternal characteristics are usually interpreted as showing that both mothers and fathers influence children’s outcomes.

 This viewpoint is theoretically appealing. However, the methodological problems that arise when this approach is empirically implemented have so far often been overlooked. From the counterfactual perspective, it is problematic to control for characteristics of fathers and mothers in the same model for the same reason that makes it problematic to control for different indicators of social origin in the same model. Paternal and maternal education, occupation, and wealth influence each other and it is not clear which of these two (or, if combined with the first research practice that includes several measures of social origin in the same model, six) variables precedes the other (Lawrence and Breen 2016). We know, however, that there is a strong tendency among men and women to form relationships with partners with similar educational and socioeconomic characteristics, a process called assortative mating (Blossfeld 2009; Schwartz 2013). In addition, partners’ can influence each other’s’ socioeconomic characteristics even after partnership formation. For instance, partners may adapt their jobs, and, as a consequence, their occupation and their wealth together. For that reason, paternal and maternal occupation (and wealth) may influence each other.

**Fig. 4:**
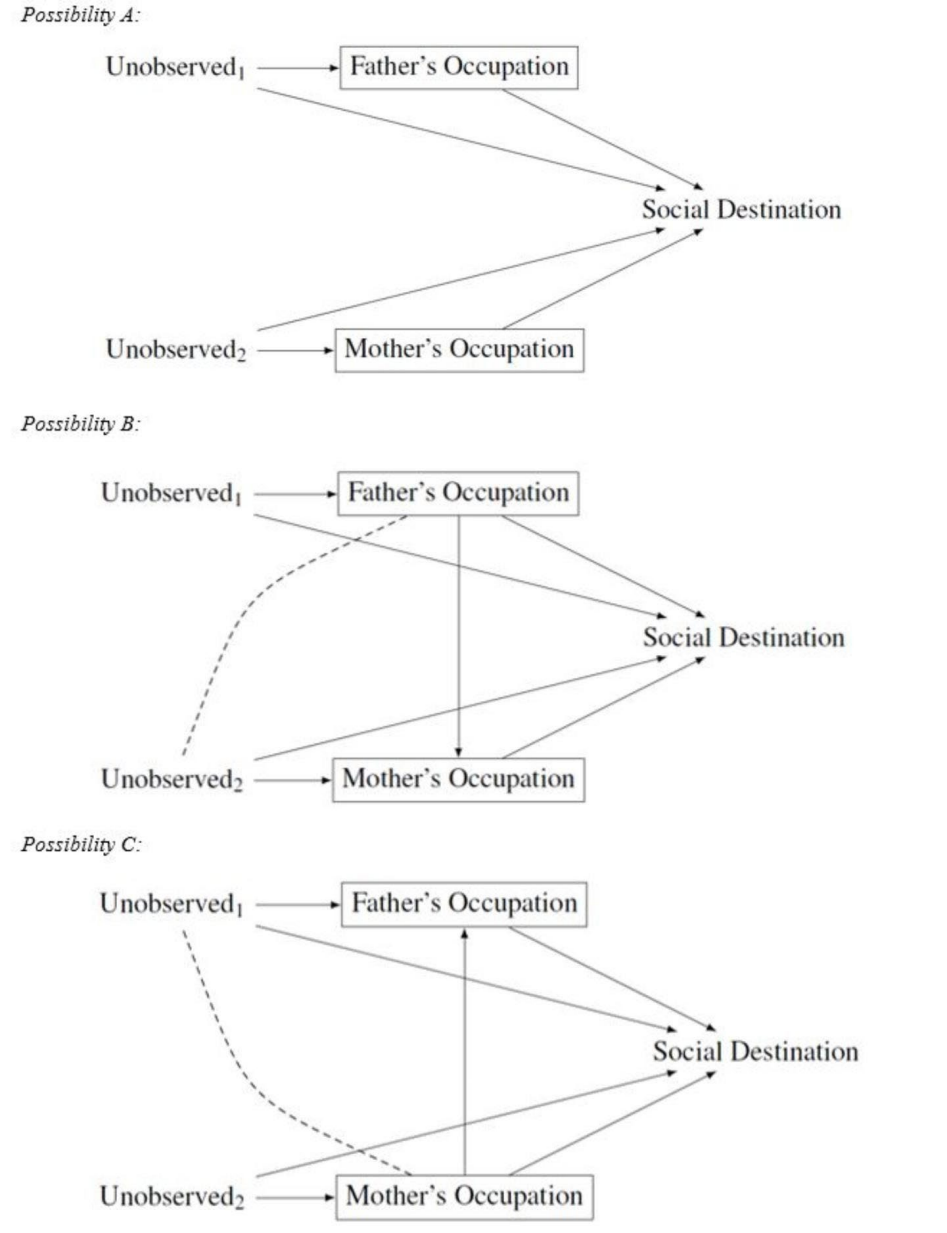
Conditioning on father’s and mother’s characteristics. Social destination: measured, for instance, via child education, income, or occupation. Unobserved_1_ and Unobserved_2_ are vectors of unobserved variables

 As an example of this research practice, Fig. 4 presents the relationships between father’s occupation, mother’s occupation, and child education. Researchers who enter father’s and mother’s occupation in the same model need to assume that they do not affect each other (Possibility A). But because of assortative mating and joint decision making of the partners this is certainly not the case. Therefore, controlling for the other parent’s occupation leads to overcontrol bias (Possibility B and Possibility C).^10^ For this reason, from a counterfactual perspective it is best not to control for the other parent’s characteristics when estimating the effects of father’s or mother’s characteristics on children’s outcomes. Otherwise, the total causal effect of any parents’ characteristic will not be identified.

 It is possible that partly the same variables are part of Unobserved_1_ and Unobserved_2_ in Fig. 4. Conditioning on the other parent’s characteristics does, however, still not improve causal inference, as even if it were to partly control for some otherwise unobserved confounders, it would still introduce overcontrol bias. The only solution would be to condition on the variables included in Unobserved_1_ and Unobserved_2_.

 It is important to point out that this critique is not a critique of the theoretical viewpoint that both fathers and mothers influence children’s outcomes but of the methods that research has so far employed to provide empirical support for this claim. Previous research did not provide any support for the claim that mothers and fathers do have different effects on children because the studies all controlled for both maternal and paternal characteristics in the same model (Beller 2009; Bloome and Western 2011; Buis 2013; Jæger 2007; Kalmijn 1994; Korupp et al. 2002; Marks 2008). The estimates obtained conditioning on the other’s parent characteristics are, however, different from the total causal effects of maternal or paternal characteristics on children’s outcomes. To test whether “mothers matter” (Beller 2009; Korupp et al. 2002) requires estimating the total effect of mothers on children. This total effect is estimated with overcontrol bias in models that include indicators of both maternal and paternal resources.

 Ideally, researchers who want to test whether mothers and fathers have different causal effects on children should analyse the effects of exogenous increases in either maternal or paternal resources in a context in which they can convincingly show that the resources of the other parent did not increase (or did only increase as a consequence of an exogenous increase in the resources of the first parent). For instance, researchers could exploit educational reforms that affected only women (or men) but had no effect on men (or women). It is only these estimates that can be used to identify the total causal effects of mothers (or fathers) on children’s outcomes. I am not aware of any study that has so far implemented such an approach.

 Again, as with respect to the first research practice, overcontrol bias is not the only concern in Fig. 4 but there is also a threat of endogenous selection bias. Let us assume we are interested in estimating the causal effect of maternal education on child education and condition on paternal education. In this scenario, probably Possibility C in Fig. 4 is the most likely data-generating process we can have in mind and paternal education is a collider variable on the path from maternal education to child education. Conditioning on the collider variable opens up a spurious association between maternal education and any unobserved variables that affect both paternal education and child education (indicated by the dashed line in Fig. 4). There are many likely candidates for such unobserved variables, in particular attributes specific to the father. In any case, researchers, who want to argue that there is no endogenous selection bias, would have to demonstrate that no such unobserved variables exist—an unlikely scenario. To avoid endogenous selection bias, we should not condition on paternal education when estimating the effect of maternal on child education. For the same reason, we should not condition on maternal education when estimating the effect of paternal on child education.

### Research practice 3: conditioning on children’s educational attainment when estimating the effect of social origin on children’s labor market outcomes

 In research on occupational mobility, the effect of social origin on social destination is often estimated conditional on educational attainment (e.g., Bernardi and Ballarino 2016; Blau and Duncan 1967; Breen 2004; Featherman and Hauser 1978; Ishida, Müller, and Ridge 1995; Sewell and Hauser 1975). This research tradition refers to the relationship between social origin, education, and social destination as the origin-education-destination (OED) triangle. Similarly, some studies investigated the role of education in income mobility (e.g., Bloome et al. 2018; Bloome and Western 2011; Gregg et al. 2017). A related topic is estimating how the effect of parental occupation on respondent’s occupation is moderated by respondent’s education. This has been done by including an interaction between parental occupation and children’s education (Hout 1988; Torche 2011). Finally, a further variation of this approach conditions on education by running analyses on a selected sample, for instance by focusing only on graduates from institutions of tertiary education (e.g., Jacob et al. 2015).

**Fig. 5:**
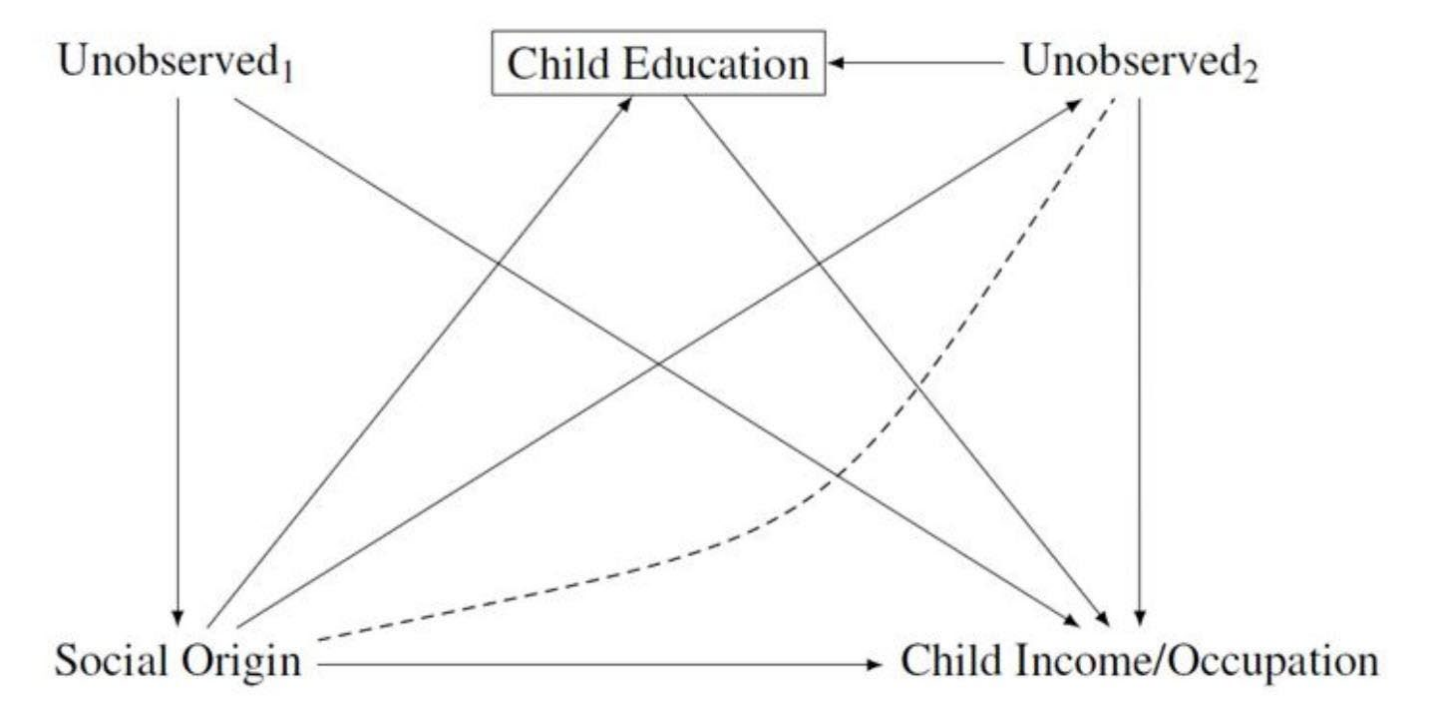
Conditioning on children’s educational attainment when estimating the relationship between social origin and children’s labor market outcomes. Social origin: measured, for instance, via parental education, income, or occupation. Unobserved_1_ and Unobserved_2_ are vectors of unobserved variables

 These three different research questions can be conceptualized through a common data-generating process. Figure 5 presents the model between social origin (parental income, education, or occupation), child education, and social destination (child occupation or income) that studies following this research practice estimate.

 The figure shows that estimating these relationships requires conditioning on a variable (child education) lying on the causal path between social origin and social destination. Admittedly, this is the purpose of this research practice: to decompose the total effect of social origin on social destination in an indirect one mediated by educational attainment and a direct one not mediated by educational attainment. In any case, there are two issues arising which affect the interpretation of these estimates.

First, the total effect of social origin is not identified in an analysis that estimates only the direct effect of social origin on social destination after conditioning on educational attainment. Certainly many researchers are aware of this issue, but it can run counter to the intuitive interpretation of research results (Acharya et al. 2016). From this point of view, the highest danger to misinterpret the estimates have studies that use a selected sample. These studies cannot estimate the total effect of social origin on social destination because they do not have the necessary information. This limitation is not always reflected in the language researchers employ. For instance, Jacob et al. (2015) spoke throughout the paper of “the impact of social origin”, even though the study did estimate only the direct effect of social origin conditional on completing a degree at an institution of tertiary education on children’s occupation but not the total effect of social origin on children’s occupation (and this is also only the case if we believe that there were no unobserved, confounding variables).

 It is also difficult to interpret the direct effect in counterfactual terms. Any change in social origin is likely to affect the process of educational attainment and therefore selection into the sample used in these studies. Without knowing the total effect, it is impossible to determine the consequences of changing social origin.

 Second, a further problematic issue in Fig. 5 is that educational attainment is a collider variable on the path from social origin to child income/ occupation. Conditioning on educational attainment opens a non-causal path from social origin to child income/ occupation via the node “Unobserved_2_” (indicated by a dashed line between “Unobserved_2_” and “social origin”). “Unobserved_2_” refers to unobserved variables that affect both educational attainment and social destination (income or occupation). There are many possible unobserved variables of this kind, for instance, respondent’s motivation, effort, and noncognitive skills. There may be an overlap between the variables included in “Unobserved_1_” and “Unobserved_2_” so that one may argue that researchers should in any case control for unobserved variables. However, there can be unobserved variables that connect educational attainment and labour market outcomes but that do not influence social origin. In any case, it is important to theoretically separate these two biases (omitted variable bias and endogenous selection bias).

 If a researcher cannot control for the variables included in “Unobserved_2_”, the resulting estimates of the direct and indirect effects will suffer from endogenous selection bias. What makes the situation even more complicated is that social origin can affect the variables included in “Unobserved_2_”. In that case, controlling for the variables included in “Unobserved_2_” will also lead to a bias, namely to overcontrol bias in estimating the direct effect of social origin on social destination. Acharya et al. (2016) called this special type of endogenous selection bias intermediate variable bias.

 The issue of endogenous selection bias is usually not dealt with or even discussed in research analysing the OED triangle. A recent exception is Zhou (2019), who controlled for a number of confounders, which lead to selection into education and which were residualized from parental income, to estimate variation in the association between parental and child income by child education. This approach, however, still relies on the assumptions of no unobserved, confounding variables “Unobserved_2_”.^11^ In sum, endogenous selection bias, provides a serious threat to the identification of the direct effect of social origin on social destination, i.e. the effect of social origin on social destination conditional on educational attainment, which researchers should be aware of.

### Research practice 4: conditioning on children’s academic performance when estimating the effect of social origin on children’s educational attainment

 A very similar triangle to the OED triangle can be drawn with respect to educational attainment as an outcome variable. This triangle reflects the, in particular among European sociologists, popular distinction between primary and secondary effects (e.g., Boudon 1973; Erikson et al. 2005; Jackson 2013; Jackson et al. 2007). A related approach, leading to the same issues of overcontrol and endogenous selection biases, is estimating the effect of social origin on social destination whilst conditioning on cognitive skills in the children’s generation (e.g., Bukodi et al. 2017; Bukodi et al. 2014; Erikson 2016).

 The idea underlying the distinction between primary and secondary effects is that social origin influences offspring’s educational attainment via two channels. First, social origin influences children’s educational performance measured through test scores or school grades (so-called primary effects). Second, social origin affects children’s educational choices (so-called secondary effects). Crucially, the effects of social origin on educational choices are identified in this research practice by estimating the effect of social origin on educational attainment conditional on academic performance.

**Fig. 6:**
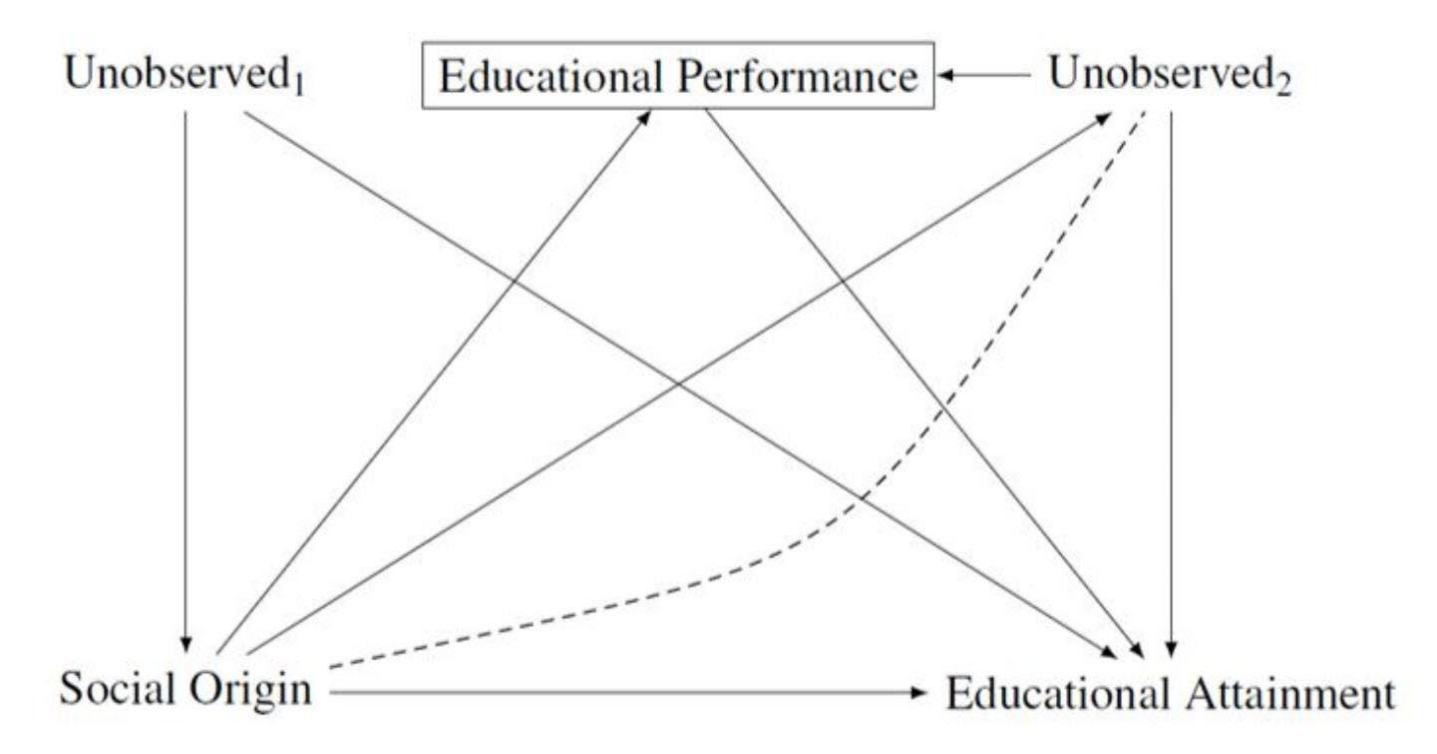
Conditioning on children’s academic performance when estimating the relationship between social origin and children’s educational attainment. Social origin: measured, for instance, via parental education, income, or occupation. Unobserved_1_ and Unobserved_2_ are vectors of unobserved variables

 Therefore, as in the other examples above, this research practice requires the researcher to condition on a variable (academic performance) lying on the causal path between the treatment (social origin) and the outcome (educational attainment) (Morgan 2012). Figure 6 presents the relationships of interest graphically. This figure is very similar to the OED triangle and, unsurprisingly, the issues of overcontrol and endogenous selection biases are in both situations the same.

 There are two important insights coming from applying the counterfactual approach to causality to this research practice. First, it is important to point out that any analysis that conditions on educational performance, i.e. that estimates the relationship shown in Fig. 6, does not estimate the total effect of social origin on children’s educational attainment. Of course, most researchers using this research practice are aware of this difference and argue that this is the aim of their analyses (Boudon 1973; Erikson et al. 2005; Jackson 2013; Jackson et al. 2007). Nevertheless, this research paradigm has affected research practices and, as in the case of the direct effect of social origin, studies that only report estimates of the direct effect, i.e. the so-called secondary effects, can be difficult to interpret.

 To give a concrete example, Dollmann (2016) estimated how a change in an educational policy in Cologne (Germany) affected educational choices. The policy changed whether parents could overrule the teacher’s recommendation for which track a child should attend in secondary school. The effect of the reform was only estimated conditional on educational performance. This practice is in line with the primary and secondary effects framework but ignores that parents can respond to policy changes by affecting children’s educational performance. Therefore, the study did not provide an unbiased estimate of the effect of the reform on any causal effect of social origin on children’s educational attainment, neither on the total effect nor on the direct effect conditional on educational performance.

To give another example, Bukodi et al. (2017) reported only estimates of the effect of social origin on social destination controlling for academic performance. This study did not provide any estimates of the total effect of social origin on children’s education. The language used in this study did, however, not reflect that they only provided estimates of the direct effect of social origin remaining after conditioning on children’s educational performance.

 Second, there is again a potential for endogenous selection bias arising from the fact that once we condition on educational performance, the analysis becomes sensitive to any unobserved variables that affect both educational performance and educational attainment. There are many likely unobserved variables of this kind, for instance, motivation, effort, and noncognitive skills. Variables that can lead to endogenous selection bias are indicated by the node “Unobserved_2_” in Fig. 6. The dashed line between between “Unobserved_2_” and “Social Origin” indicates the non-causal path between these two variables opened up by conditioning on the collider “Educational Performance”.

 The omission of variables included in “Unobserved_2_” provides a likely source of bias in the estimation of the direct effect of social origin on educational attainment net of educational performance. At the same time, some of the variables included in “Unobserved_2_” may be affected by social origin and controlling for them would lead to overcontrol bias, i.e. we have the situation which Acharya et al. (2016) called intermediate variable bias. For this reason, other, more direct ways to identify the effect of social origin on educational decision-making are needed to provide support to the model of primary and secondary effects (Morgan 2012).

## Data and methods

### Data

 In the empirical part of this article, I quantify the consequences of overcontrol bias for our conclusions about intergenerational mobility. In order to do so, I employ data from the German Socio-Economic Panel Study (SOEP; Goebel et al. 2018). These data are widely used to study intergenerational mobility in Germany (e.g., Grätz and Pollak 2016; Hertel 2017; Müller and Pollak 2004). The sample I use includes male and female respondents born between 1970 and 1998. They are the (adult) children’s generation to whom parental characteristics are added.

### Variables

 I describe shortly the variables used in the empirical analysis. I use simple continuous or dummy variables of the concepts of interest. Measures that are more complex could be constructed but as the aim of the empirical analysis is to illustrate the consequences of overcontrol and endogenous selection biases, the variables I employ are sufficient and allow me a straightforward discussion of the important issues. Whilst I acknowledge that issues of measurement are important (and much attention in the intergenerational mobility literature is devoted to them), they are not the focus of this article. The issues of overcontrol and endogenous selection biases arise independently of the way in which social origin and social destination are measured.

*Occupational Status.* Occupation in the children’s generation is the central outcome variable in research on occupational mobility. I employ a continuous variable of occupational status based on the International Socio-Economic Index of Occupational Status (ISEI; Ganzeboom, de Graaf, and Treiman 1992). This measure is standardized to have a mean of 0 and a standard deviation of 1.

*Educational Attainment.* I measure educational attainment in the children’s generation through years of education. This variable is the outcome variable in studies on educational mobility. In addition, many studies on occupational mobility use this variable as a control variable to estimate the effect of social origin on social destination conditional on educational attainment in the children’s generation.

*Social Origin.* I employ three measures of social origin. These variables are based on parental characteristics of the respondents. I measure father’s and mother’s occupation. Father’s and mother’s occupation refer to the occupational status (measured again via ISEI) of the respective parent. I standardize father’s and mother’s occupation to have a mean of 0 and a standard deviation of 1. In addition, I measure father’s education. This variable is coded as 1 if the father of the respondent holds an *Abitur* degree (a high school certificate and requirement to study at university) and as 0 for all lower educational degrees. These three measures of social origin allow me to discuss all issues raised in the theoretical considerations. Further extensions (e.g., to include measures of parental wealth or parental income) could easily be applied with similar implications.

*Cognitive Skills.* To discuss the consequences of including measures of academic performance in models predicting educational attainment, I employ a measure of cognitive skills that is based on a test that was conducted as part of the survey when the respondents were 16 to 17 years old.

*Gender.* In all models, I control for respondent’s gender by including a dummy variable for male (adult) children.

 Descriptive statistics on all variables used in the analysis are reported in Table 1.

**Table 1:** Descriptive statistics

Variable	Mean	SD	Min	Max	*N*
Male	0.45	0.50	0	1	4,827
Occupational status (standardized)	0.00	1.00	− 1.85	2.62	4,827
Years of education	12.97	2.71	7	18	4,827
Cognitive skills (standardized)	0.00	1.00	− 3.11	2.33	471
Father’s occupational status (standardized)	0.00	1.00	− 1.62	2.75	4,827
Mother’s occupational status (standardized)	0.00	1.00	− 1.66	2.98	4,827
Father *Abitur*^1^ (high level of education)	0.20	0.40	0	1	4,827

Notes: *Abitur* is the highest secondary school leaving certificate in the German education system

Source: German Socio-Economic Panel Study (SOEP), v36 (DOI: 10.5684/soep.v36)

### Analytic strategy

 I rely on OLS regression analysis, the standard tool to estimate intergenerational mobility. Sometimes, researchers use other techniques to estimate intergenerational mobility, such as log-linear modelling. However, all the issues I discuss in this article do occur in these models as well. Focusing on the linear case allows me to keep the technical aspects simple and to focus on the conceptual issues that I emphasize in this article.

## Results

 In the following, I report a set of regression models that illustrate the consequences of employing the four research practices discussed in this article. The aim of this exercise is to demonstrate that the overcontrol bias introduced by these practices is not just a hypothetical scenario but that this bias is substantive in size and affects our conclusions about intergenerational mobility. The size and direction of endogenous selection bias cannot be determined.^12^ It goes without saying that all estimates reported here may suffer from omitted variable bias; the focus of this article is not on addressing omitted variable bias but on bringing the largely ignored issues of overcontrol and endogenous selection biases to the attention of researchers working on intergenerational mobility.

 As in the theoretical considerations, I start by discussing the issue of including several indicators of family background in the same model. In Tables 2, I present models predicting both respondent’s occupational status and their educational attainment, measured through years of education. The different models show what happens when different indicators of social origin are included in separate models (Models 1 and 2 as well as Models 4 and 5) as well as when these measures are entered in the same models (Models 3 and 6).

**Table 2:** OLS regression models predicting occupational status and educational attainment

	Occupational status (ISEI)			Educational attainment (Years of education)		
	(1)	(2)	(3)	(4)	(5)	(6)
Father’s occupational status	0.34^**^		0.30^**^	1.07^**^		0.79^**^
	[0.32, 0.37]		[0.26, 0.33]	[1.00, 1.14]		[0.71, 0.88]
Male	–0.01	0.01	− 0.01	− 0.40^**^	− 0.33^**^	− 0.37^**^
	[− 0.06, 0.04]	[− 0.05, 0.06]	[− 0.06, 0.05]	[− 0.54, − 0.26]	[− 0.47, − 0.19]	[− 0.51, − 0.23]
Father *Abitur* (high level of education)		0.63^**^	0.22^**^		2.34^**^	1.23^**^
		[0.56, 0.70]	[0.14, 0.30]		[2.16, 2.52]	[1.02, 1.44]
*N*	4,827					
R^2^	0.119	0.064	0.124	0.161	0.125	0.184

Note: 95% confidence intervals in brackets

Source: SOEP v36 (DOI: 10.5684/soep.v36)

^*^ *p* < 0.05, ^**^ *p* < 0.01

 I focus in the interpretation of the findings on how the estimates of the effects of different indicators of social origin on adult children’s outcomes change when other indicators of social origin are added to the same model. For instance, Model 2 shows that having a father with a high level of parental education (an *Abitur* degree) leads to a 0.63 standard deviations higher occupational status in the children’s generation. The effect is, however, only about one third of this size (0.22) once we control for father’s occupational status (Model 3). This comparison shows that the overcontrol bias introduced by controlling for two measures of social origin in the same model affects the amount of mobility observed. As in this example, overcontrol bias due to controlling for several indicators of social origin leads usually to an underestimation of the effect of social origin on children’s occupational status. This is the case along as all indicators of social origin are positively correlated with each other and with the outcome. This is usually the case.

 The conditional estimate (Model 3) is likely to suffer not only from overcontrol but also from endogenous selection bias. Contrary to overcontrol bias, it is, however, not possible to say in which direction endogenous selection bias may influence the estimates (Acharya et al. 2016; Breen 2018). Because of endogenous selection bias, Model 3 does also not identify the direct effect of father’s education conditional on father’s occupation.

 Using educational attainment as an outcome variable (Models 4 to 6) instead of occupational status leads to very similar results. Overcontrol bias introduced by a control for father’s occupational status (Model 6) leads us to underestimate the total effect of parental on child education (Model 5) and the size of the endogenous selection bias (Model 6) cannot be quantified.

 It is important to keep in mind that many studies only report the model that includes several indicators of social origin, i.e. Models 3 and 6 in my example. For this reason, these studies do not allow the reader to identify the size of the overcontrol bias.

 The second research practice I discuss in this article is the inclusion of measures of maternal and paternal characteristics in the same model. In Tables 3, I show models that predict occupational status and years of education. The models compare results if father’s and mother’s characteristics are entered into separate (Models 1 and 2 as well as Models 4 and 5) and into the same models (Models 3 and 6).

**Table 3:** OLS regression models predicting occupational status and educational attainment

	Occupational status (ISEI)			Educational attainment (Years of education)		
	(1)	(2)	(3)	(4)	(5)	(6)
Father’s occupational status	0.34^**^		0.27^**^	1.07^**^		0.86^**^
	[0.32, 0.37]		[0.24, 0.30]	[1.00, 1.14]		[0.78, 0.94]
Male	− 0.01	− 0.00	− 0.01	− 0.40^**^	− 0.37^**^	− 0.40^**^
	[− 0.06, 0.04]	[− 0.06, 0.05]	[− 0.06, 0.04]	[− 0.54, − 0.26]	[− 0.51, − 0.22]	[− 0.53, − 0.26]
Mother’s occupational status		0.29^**^	0.18^**^		0.87^**^	0.51^**^
		[0.26, 0.32]	[0.15, 0.21]		[0.79, 0.94]	[0.43, 0.58]
*N*	4,827					
R^2^	0.119	0.084	0.145	0.161	0.107	0.190

Note: 95% confidence intervals in brackets

Source: SOEP v36 (DOI: 10.5684/soep.v36)

^*^ *p* < 0.05, ^**^ *p* < 0.01

 Again, I interpret the results from the perspective of the counterfactual approach to causality. Of course, there are many unobserved variables that are likely to confound the relationship between the measures of social origin and the measures of social destination so that it is impossible to argue that these estimates identify causal effects. However, my argument is that Model 2 presents a better estimate of the total effect of maternal on child occupational status (0.29) than the smaller estimate obtained after introducing overcontrol and endogenous selection biases through conditioning on paternal occupation in Model 3 (0.18). Similarly, the total effect of paternal occupational status on child education is 1.07 in Model 4 but it is underestimated in Model 6 (0.86), which conditions on maternal occupational status. To conclude, as with respect to the first research practice, the practice to include measures of maternal and paternal characteristics in the same model leads to downwardly biased estimates of intergenerational persistence because of overcontrol bias. These biases are substantively large in size and affect our conclusions about intergenerational mobility. The size of the endogenous selection bias can, however, not be identified (Acharya et al. 2016; Breen 2018).

 The third research practice, which I illustrate here, is the estimation of the effects of family background on occupational outcomes conditional on education. In Tables 4, the so-called origin—education—destination (OED) triangle is estimated. Model 1 estimates the effect of father’s on adult children’s occupational status. Model 2 estimates this effect conditional on respondent’s educational attainment.

**Table 4:** OLS regression models predicting occupational status

	Occupational status (ISEI)	
	(1)	(2)
Father’s occupational status	0.34^**^	0.12^**^
	[0.32, 0.37]	[0.09, 0.14]
Male	− 0.01	0.07^**^
	[− 0.06, 0.04]	[0.03, 0.12]
Years of education		0.21^**^
		[0.20, 0.22]
*N*	4,827	
R^2^	0.119	0.392

Note: 95% confidence intervals in brackets

Source: SOEP v36 (DOI: 10.5684/soep.v36)

^*^ *p* < 0.05, ^**^ *p* < 0.01

 The effect of father’s on child occupational status is 0.34 (Model 1). It is reduced once we control for educational attainment to 0.12 (Model 2). It is very difficult to interpret this conditional estimate. In any case, Model 2 alone does not allow us to say anything about the total causal effect of father’s occupation on child occupation. Studies that only present the estimate of Model 2 should therefore be interpreted with caution.

 As discussed in the theoretical considerations, it is also difficult to argue that 0.12 is the “direct effect” (Bernardi and Ballarino 2016) of father’s on child occupation as conditioning on education open ups the possibility of endogenous selection bias. There are many possible candidates for such unobserved variables, such as ability and motivation, which may affect both educational attainment and occupational status and therefore confound the estimation of the “direct effect” of social origin on occupational status. It is impossible to say in which direction endogenous selection bias influences the estimates and how large this bias is (Acharya et al. 2016; Breen 2018).

 Finally, the last research practice that I discuss is to condition on academic performance when estimating the association between family background and child education. In Tables 5, I present a simple form of estimates of the so-called “primary” and “secondary effects” (Boudon 1973; Erikson et al. 2005; Jackson 2013; Jackson et al. 2007). Model 1 shows the (gross) association between father’s occupational status and respondent’s education. Model 2 shows this relationship after conditioning on children’s cognitive skills, a measure of academic performance, providing an estimate of secondary effects.

**Table 5:** OLS regression models predicting educational attainment

	Educational attainment (Years of education)	
	(1)	(2)
Father’s occupational status	0.80^**^	0.55^**^
	[0.61, 0.99]	[0.37, 0.74]
Male	− 0.37	− 0.51^**^
	[− 0.78, 0.03]	[− 0.88, − 0.13]
Cognitive skills		0.89^**^
		[0.70, 1.09]
*N*	471	
R^2^	0.131	0.260

Note: 95% confidence intervals in brackets

Source: SOEP v36 (DOI: 10.5684/soep.v36)

^*^ *p* < 0.05, ^**^ *p* < 0.01

 As expected, the effect of father’s occupation on child education is smaller (0.55; Model 2) once we control for cognitive skills than without controlling for this variable lying on the causal path from father’s occupation to child education (0.80; Model 1). Again, the question is how this conditional estimate can be interpreted. Certainly it should not be confused with the total causal effect of social origin on educational attainment, which is estimated (under the strong assumption of no unobserved, confounding variables) by Model 1 and not by Model 2.

 In addition, even this estimate of the direct effect obtained in Model 2 can be confounded by the endogenous selection bias introduced through conditioning on children’s cognitive skills. This bias can invalidate any claim that the conditional estimate provides a representation of a “direct effect” of social origin on children’s educational attainment after conditioning on children’s academic performance. In addition, the direction and the size of this bias cannot be foreseen (Acharya et al. 2016; Breen 2018). For this reason, Morgan (2012) argued that other ways were needed to identify the influence of socioeconomic differences in educational decision-making on children’s educational attainment.

## Discussion and conclusion

 The emergence of the counterfactual approach to causality has affected research practices in the social sciences. However, there are some widely used research practices that do not take into account the lessons that we can learn from this improved understanding of causality. This article discusses four of these practices, which are taken from the field of research on the intergenerational transmission of advantage.

 Readers of this article may believe that the main message of it is negative, as it demonstrates several types of biases arising through widely spread research practices and as it argues that it is difficult to overcome these biases. However, faced with these difficulties, I am convinced it is important to point out the importance of descriptive estimates. The arguments presented in this article underline the importance of gross estimates of the associations between social origin and social destination for research on intergenerational mobility (Torche 2015). These bivariate associations are the main contribution intergenerational mobility research makes to our understanding of contemporary and historic societies. Researchers have to be aware that conditioning on other variables often complicates identification and makes it harder to understand what models are actually estimating. What is more, conditioning on variables can lead to overcontrol and endogenous selection biases. Apart from the four research practices that I discuss in this article, there are other research practices than can lead to overcontrol and endogenous selection biases. For instance, Torche (2015) discussed the problems in interpreting mechanisms underlying the intergenerational transmission of advantage.

 The issues identified in this article have general significance for the way research on intergenerational mobility is conducted. For instance, mobility research is often interested in estimating variation in the effect of social origin on social destination over time. In this context, the overcontrol and endogenous selection biases introduced by the four research practices discussed in this article are very consequential. If researchers condition in the same model on different indicators of social origin and/ or on maternal and paternal characteristics (e.g., Beller 2009; Bloome and Western 2011; Duncan et al. 2017; Mare 1981; Shavit and Blossfeld 1993), they can find indications of change in intergenerational mobility even if the effect of every single indicator of social origin on social destination did not change. This can simply happen because the associations between the different indicators of social origin and/ or between paternal and maternal characteristics can change over time. However, that is certainly not what we mean if we talk about changing effects of social origin on social destination over time or across cohorts. It is therefore recommendable if research estimating changes in intergenerational mobility across cohorts focuses on the gross estimates of the associations between one indicator of social origin and a measure of social destination without conditioning on other measures of social origin (e.g., Breen et al. 2009).

 Similar arguments apply to comparisons across countries. If researchers condition on several indicators of social origin in these comparisons, they can observe differences in the effects of one indicator of social origin on children’s outcomes even if the effect of this indicator on children’s outcomes does not vary across countries. This can happen if the associations between different indicators of social origin vary across countries. Again, my main recommendation is to focus on the gross estimates and to compare these across countries.

 If researchers insist on employing the first two research practices discussed in this article, it is a good practice if they report the gross estimates without conditioning in addition to the conditional, net estimates (e.g., Duncan et al. 2017; Mood 2017; Pfeffer 2018). By these means, readers can at least see the initial, gross estimates for themselves and can interpret the results taking them into account. Some research reports only the conditional estimates making it impossible for the reader to assess the impact of overcontrol and endogenous selection biases.

 Recommendations with respect to the third and the fourth research practice are more difficult. Elwert and Winship (2014) recommend not to condition on collider variables. This recommendation is, however, not very helpful for researchers who would like to employ these research practices, as conditioning on collider variables is necessary to answer the research questions that motivate these research practices. In statistics, epidemiology, and political science, a field of causal mediation analysis has been developing in the last decades (e.g., Acharya et al. 2016; Imai et al. 2011; Pearl 2009; Robins and Greenland 1992; VanderWeele 2015). These researchers have been developing traditional mediation analysis (Baron and Kenny 1986) further trying to address the biases discussed in this article. Some sociologists have integrated these insights in their work (e.g., Brand et al. 2019; Breen et al. 2013; Lawrence and Breen 2016; Song 2016; Wodtke et al. 2011; Zhou and Wodtke 2019) and these approaches could also be applied to research on intergenerational mobility (as in Zhou 2019). However, it is important to note that these approaches still rely on measuring all unobserved, confounding variables.^13^ Of course, there are approaches to obtain causal estimates which allow researchers to control for unobserved variables (see for instance the section on “intergenerational effects” in Björklund and Jäntti [2020]). However, these approaches usually only identify total causal effects and not the direct effects on which much attention in the sociological literature on intergenerational mobility has focused.

 Generally, I think the most important message of this article is that researchers working on the intergenerational transmission of advantage need to reflect upon the possibilities of overcontrol and endogenous selection biases in their research. There may be good reasons to employ any of the four research practices discussed in this article but at least researchers should be aware of the pitfalls connected to them.

## Data Availability

The data used in the manuscript is available via the German Socio-Economic Panel Study (SOEP) at the German Institute for Economic Research (DIW), Berlin.

## Footnotes

1 For this reason a causal language is used throughout this article. Sometimes researchers avoid the use of causal language when employing the four research practices discussed below. However, these practices do require a causal interpretation and therefore it is appropriate to use a causal language when employing them, whilst being aware of the threats to causal inference caused by omitted variable, overcontrol, and endogenous selection biases (see also Hernán 2018).

2 Conditioning on variables can be done in different ways, for instance, by adding these variables as control variables or by using these variables to select the sample used in an analysis (Elwert and Winship 2014). The issues of overcontrol and endogenous variable biases are in all situations the same.

3 The total causal effect is also called the average treatment effect (ATE) (e.g., Acharya et al. 2016).

4 A descendant is a variable following on the causal path from another variable. A descendant of a collider is a variable causally affected by a collider.

5 It is important to keep in mind that a model that conditions on a mediator does not provide an estimate of the total causal effect. To obtain this estimate of the total causal effect, it is necessary to estimate a model that does not condition on the mediator.

6 We can also talk about the causal path A → B.

7 As mentioned above, to some degree this reasoning could also apply to the relationship between parental wealth and parental education which is why it may also lead to overcontrol bias if researchers control for parental wealth when estimating the effect of parental education on social destination.

8 In most applications in research on intergenerational mobility and in the empirical example I report below different measures of social origin are measured at the same time point. Often respondents provide retrospective information on their parents’ resources during the childhood of the respondents. In the case that data are measured at different time points, causality may be more likely to be inferred based on the rule that a cause has to proceed an effect. However, it is important to distinguish the measured occurrence of different parental resources and their actual occurrence. Even if parental occupation is measured at an earlier time point than parental wealth, parental wealth could causally proceed parental occupation, and vice versa.

9 There is also a possibility that in research practices 1 and 2, similar to as discussed for research practices 3 and 4, endogenous selection bias takes the form of intermediate variable bias (Acharya et al. 2016). This could be visualized through arrows running from parental education (and, depending on which of the three possibilities in Fig. 3 is preferred also from parental occupation or parental income) to “Unoberserved_2_” and “Unobserved_3_”. I did not include these arrows in Fig. 3 (and in Fig. 4), as these figures are already very complex.

10 A possible way to take into account the characteristics of both parents and to avoid overcontrol and endogenous selection biases is to construct a single measure combining information from both parents. For instance, Hout (2018) constructed occupational scores using information on both fathers’ and mothers’ occupations and weighting these two components. Such an approach can be applied if researchers are interested in estimating the joint effect of maternal and paternal characteristics on children but it does not allow them to isolate the effects of mothers or fathers, which is the main motivation of the “mothers matter” (Beller 2009; Korupp et al. 2002) literature.

11 Zhou (2019:477) acknowledges this: “our estimation of controlled mobility rests on the assumption that we have adjusted for all covariates that may lead to selection bias.”

12 As Breen (2018: 608) wrote: “The first problem discussed here, of collider bias in the estimation of direct effects, involves unobserved variables, and so it is impossible to know the magnitude or direction of the bias (if any) in any specific study. We can say something about the bias under certain assumptions, but these include assumptions about unobserved relationships and so cannot be tested.”

13 The literature on sensitivity analysis aims to quantify how large the bias through unobserved variables has to be to change conclusions based on selection on observed variables approaches (Hong et al. 2018; Imai et al. 2010; Imbens 2003; VanderWeele 2010; VanderWeele and Chiba 2014). However, it remains unknown in most applications in the social sciences how large the bias through unobserved variables actually is. Therefore, it remains often unclear whether the actual bias is close or far away from the bias needed to affect the conclusions of an analysis that relies on a selection on observed variables approach.

## Code availability

The do-files to replicate the analysis are available upon request.
